# Challenges of scene management in road traffic accidents: a systematic literature review

**Published:** 2019-08

**Authors:** Reyhaneh Mostafanejad, Ali Janati, Homayon Sadeghi-Bazargani, Javad Babaie

**Affiliations:** ^*a*^Health Services Management Department, Faculty of Management and Medical Information, Tabriz University of Medical Sciences, Tabriz, Iran.; ^*b*^Iranian Center of Excellence in Health Management, Department of Health Services Management, School of Management and Medical Informatics, Tabriz University of Medical Sciences, Tabriz, Iran.; ^*c*^Department of Statistics and Epidemiology, School of Health and Nutrition, Tabriz University of Medical Sciences, Tabriz, Iran.; ^*d*^Department of Health Services Management, Iranian Center of Excellence in Health Management, School of Management and Medical Informatics, Tabriz University of Medical Sciences, Tabriz, Iran.

## Abstract

**Background:**

Road Traffic Accidents (RTAs) are among important causes of mortality worldwide. Managing RTA scenes is in turn a vital factor in reducing subsequent mortalities and injuries. Therefore, the present study aims to identify the challenges of Scene Management (SM) in RTAs.

**Methods:**

The indexed literature in international databases including Medline, Scopus, Web of Science and search engines including Google scholar, Pubmed and also Iranian databases of Irandoc, Iranmedex, Magiran, and SID were systematically searched using traffic accidents and scene management as keywords. The extracted studies then were screened and finally 13 papers were included.

**Results:**

The challenges extracted from the investigated studies were classified into 6 categories including: difficulty of the nature of scene management and taking responsibility in it, lack of coordination, managing bystanders and volunteers, insufficient equipment and supplies, infrastructure deficiencies, and involved staff’s insufficient skills.

**Conclusions:**

SM is a challenging process in the world and almost managed poorly worldwide. Some of these difficulties stem from the challenging nature of SM in RTAs. However, a great bulk of the challenges can be resolved through training, providing equipment and infrastructure, and more inter-sectional coordination.

**Keywords:**

Road traffic accidents, Scene management, Mortality, Response

